# Ferroptosis-Related Genes in Bronchoalveolar Lavage Fluid Serves as Prognostic Biomarkers for Idiopathic Pulmonary Fibrosis

**DOI:** 10.3389/fmed.2021.693959

**Published:** 2021-10-04

**Authors:** Meng Li, Ke Wang, Yanpeng Zhang, Meng Fan, Anqi Li, Jiejun Zhou, Tian Yang, Puyu Shi, Dan Li, Guangjian Zhang, Mingwei Chen, Hui Ren

**Affiliations:** ^1^Department of Respiratory and Critical Care Medicine, The First Affiliated Hospital of Xi'an Jiao Tong University, Xi'an, China; ^2^Department of Talent Highland, The First Affiliated Hospital of Xi'an Jiao Tong University, Xi'an, China; ^3^Department of Thoracic Surgery, The First Affiliated Hospital of Xi'an Jiao Tong University, Xi'an, China; ^4^Department of Center for Translational Medicine, The First Affiliated Hospital of Xi'an Jiao Tong University, Xi'an, China

**Keywords:** idiopathic pulmonary fibrosis, ferroptosis, prognostic, signature, model

## Abstract

**Background:** Idiopathic pulmonary fibrosis (IPF) is a chronic progressive disease with unknown etiology and unfavorable prognosis. Ferroptosis is a form of regulated cell death with an iron-dependent way that is involved in the development of various diseases. Whereas the prognostic value of ferroptosis-related genes (FRGs) in IPF remains uncertain and needs to be further elucidated.

**Methods:** The FerrDb database and the previous studies were screened to explore the FRGs. The data of patients with IPF were obtained from the GSE70866 dataset. Wilcoxon's test and univariate Cox regression analysis were applied to identify the FRGs that are differentially expressed between normal and patients with IPF and associated with prognosis. Next, a multigene signature was constructed by the least absolute shrinkage and selection operator (LASSO)-penalized Cox model in the training cohort and evaluated by using calibration and receiver operating characteristic (ROC) curves. Then, 30% of the dataset samples were randomly selected for internal validation. Finally, the potential function and pathways that might be affected by the risk score-related differently expressed genes (DEGs) were further explored.

**Results:** A total of 183 FRGs were identified by the FerrDb database and the previous studies, and 19 of them were differentially expressed in bronchoalveolar lavage fluid (BALF) between IPF and healthy controls and associated with prognosis (*p* < 0.05). There were five FRGs (aconitase 1 [ACO1], neuroblastoma RAS viral (v-ras) oncogene homolog [NRAS], Ectonucleotide pyrophosphatase/phosphodiesterase 2 [ENPP2], Mucin 1 [MUC1], and ZFP36 ring finger protein [ZFP36]) identified as risk signatures and stratified patients with IPF into the two risk groups. The overall survival rate in patients with high risk was significantly lower than that in patients with low risk (*p* < 0.001). The calibration and ROC curve analysis confirmed the predictive capacity of this signature, and the results were further verified in the validation group. Risk score-related DEGs were found enriched in ECM-receptor interaction and focal adhesion pathways.

**Conclusion:** The five FRGs in BALF can be used for prognostic prediction in IPF, which may contribute to improving the management strategies of IPF.

## Introduction

Idiopathic pulmonary fibrosis (IPF) is a chronic, progressive, age-related interstitial pulmonary disease (ILD) with unknown etiology, significant morbidity, and unfavorable survival (1, 2). The prevalence of IPF is on the rise worldwide and about 40,000 new cases of IPF are diagnosed per year in Europe alone (3, 4). The life expectancy of patients with IPF is similar to that of patients with lung cancer, with median survival rates of 50 and 20% at 3- and 5-years after diagnosis, respectively (5, 6), which imposed a substantial socio-economic burden on the world (7). There was no medicine authorized for this devastating disease before 2010. Two medications, nintedanib and pirfenidone, have been proven to be safe and effective in slowing disease progression in recent years (8). Despite the advances in treatment technology, there is still no cure for IPF, and neither nintedanib nor pirfenidone improves the pulmonary function and living quality, and both the therapies have tolerability issues (9, 10). In addition, IPF was found at increased risk of severe coronavirus disease 2019 (COVID-19) due to sharing the same risk factors for severe COVID-19 (11), and as an independent risk for the development of lung cancer (12). Hence, as a devastating disease with unknown etiology and difficult treatment, and the risk factor of other fatal diseases, there is an additional need to investigate the novel biomarkers for its diagnosis, treatment, and prognosis.

Ferroptosis, a newly identified form of iron-dependent cell death unlike other cell death forms, is primarily driven by the iron excessive accumulation, lipid peroxidation, and subsequent plasma membrane rupture, which ultimately caused cell death and is often accompanied by the disturbance of lipid and iron metabolism (13). Ferroptosis has been verified to play a crucial part in the development and disease of organisms (14). Meanwhile, many genes have also been defined as markers or regulatory factors of ferroptosis [also known as ferroptosis-related genes (FRGs)] and are involved in the occurrence and development of various benign and malignant lung diseases, such as chronic obstructive pulmonary disease (COPD) (15), acute lung injury (16), asthma (17), and lung cancer (18). It is worth noting that the disturbance of certain molecular mechanisms related to metabolism and fibrosis, such as oxidative stress, lipid, and iron metabolism, in the bronchoalveolar lavage fluid (BALF) of IPF patients have been investigated in several studies recently (19–21). Elevated iron levels have been detected in the lung tissue of patients with IPF, and iron accumulation is associated with significant increases in airway fibrosis and altered pulmonary function (22). In addition, increased numbers of iron-laden macrophage clusters in BALF enable cause macrophages to produce reactive oxygen species (ROS) and result in lipid peroxidation in IPF (23). These findings indicated the potential role of ferroptosis in the pathogenesis of IPF due to the disturbance of lipid and iron metabolism contributes to the occurrence of ferroptosis. Li X et al. further elucidated that the radiation-induced lung fibrosis was significantly alleviated by ferroptosis inhibitor (24). Erastin, an inducer of ferroptosis, has been verified to promote the main pathogenesis of IPF called fibroblast-to-myofibroblast differentiation, and this effect can be suppressed by the Fer-1 (an inhibitor of ferroptosis) (25, 26). In addition, Tsubouchi K demonstrated that lipid peroxidation regulated by GPX4 is involved in the pathogenesis of IPF (27). However, no studies have been conducted on the relationship between ferroptosis and the prognosis of IPF, and the prognostic value of FRGs in IPF remains largely unknown.

Herein, we aimed to investigate whether FRGs in BALF are associated with the prognosis of IPF. First, we evaluated the expression levels of FRGs in BALF of IPF patients by downloading the mRNA expression profiles and corresponding clinical data from the Gene Expression Omnibus (GEO). Subsequently, a multigene prognostic model with FRGs was constructed and validated in the training and testing cohort, respectively. Finally, a functional enrichment analysis was performed to investigate the potential biological functions and signaling pathways affected by these risk-related differently expressed genes (DEGs). We hope to find some biomarkers that can contribute to the diagnosis, treatment, or prognosis of IPF in these ways.

## Materials and Methods

### Exploration of FRGs

The FerrDb database (http://www.zhounan.org/ferrdb/) is the first ferroptosis-related database that integrates the latest makers and regulators of ferroptosis and ferroptosis-disease associations (28). A total of 183 FRGs were identified by the Driver, Suppressors, and Marker model of the FerrDb database and the previous studies (14, 29–31). The details of FRGs are shown in Supplementary Table 1.

### Data Collection

The GSE70866 dataset is the only one that simultaneously includes the prognostic data and mRNA expression profiles in BALF of IPF (20 healthy controls and 176 IPF patients), which were obtained from GEO (https://www.ncbi.nlm.nih.gov/geo/) and normalized using the R “limma” package. This study does not need to be granted by the local ethics committee as the data of GEO are publicly available, and we strictly adhere to the data access policies and publication guidelines of the GEO. The clinical information of the GSE 70866 cohort is shown in Supplementary Table 2.

### Identification of FRGs

The mRNA expression profiles of FRGs were extracted and the DEGs of FRGs in BALF between normal and IPF were identified by the “limma” package of R with the criteria of a false discovery rate (FDR) <0.05 and *P* < 0.05. The results were visualized by the “heatmap” package.

### Identification of the Prognostic-Related FRGs

A univariate Cox regression of the “survival” package was conducted to investigate the FRGs associated with the prognosis of IPF in the GEO database. The value of *p* < 0.05 was confirmed as the filtering criteria. The results were visualized by the forest map. Next, a Venn diagram was performed to identify the intersection of DEGs of FRGs and prognostic FRGs.

### Protein–Protein Interaction (PPI) Network Construction

The protein–protein interaction (PPI) network for the overlapping genes was constructed using the STRING database (https://www.string-db.org) (32) to explore the interactions between these genes.

### Construction and Internal Validation of the Prognostic FRGs Signature

The patients with IPF from the GSE70866 dataset were randomly separated into two groups at a ratio of 7:3, the former for the training group and the latter for internal validation. The least absolute shrinkage and selection operator (LASSO)-penalized Cox regression analysis was utilized in the present study to establish a prognostic model in the training group to minimize the risk of overfitting by using the “glmnet” package of R (33, 34). First, the range of 19 FRGs associated with prognosis was narrowed through 10-fold cross-validation by using the LASSO algorithm with penalty parameter adjustment, and the datasets were sub-sampled 1,000 times and the FRGs repeated >900 times were chosen for the next analysis. Then, eight FRGs with non-zero regression coefficients were retained for the multivariate Cox regression analysis. Finally, the risk scores of the patients were calculated according to the normalized expression levels and the corresponding regression coefficients of the five FRGs, and patients with IPF in the training group were classified into high- and low-risk groups depending on the median risk score.

The “rms” package of R was utilized to visualize the risk score in the nomogram and generate the calibration curve to assess the prediction accuracy of the nomogram. Principal component analysis (PCA) and t-distributed stochastic neighbor embedding (t-SNE) were carried out with the “Rtsne” and “ggplot2” R package to explore the distribution of the two risk groups. Then, the “survival,” “suvminer,” and “timeROC” R package were utilized to generate Kaplan–Meier (K–M) survival and time-dependent receiver operational characteristic (ROC) curves to further evaluate the predictive capacity of the prognostic model, respectively. Additionally, the “heatmap” package was performed to visualize the gene expression levels in each sample in the two risk groups. Subsequently, the “survival” package was performed to evaluate whether clinical parameters and risk scores are independent prognostic factors for overall survival (OS). Finally, the results were further identified in the internal validation group.

### Functional Enrichment Analysis

The patients with IPF were classified as the high- and low-risk group base on the median risk score in the training and test cohort, and the risk-related DEGs between the two risk groups were screened out as much as possible with FDR <0.05 and |log2FC| > 0.3 in the two cohorts by using the “limma” package. Then, Gene Ontology (GO) as well as Kyoto Encyclopedia of Genes and Genomes (KEGG) analysis were performed by the “clusterProfiler” package to explore the biological functions and signaling pathways affected by the risk-related DEGs with the criteria of FDR <0.05 and *p* < 0.05 (35).

### Expression of the Prognostic Model FRGs in the Lung Tissue of IPF

The datasets containing the mRNA expression profiles of IPF lung tissue were retrieved from the GEO database. Four datasets (GSE110147, GSE72073, GSE53845, and GSE24206) with a total of 28 healthy control and 84 patients with IPF were selected for analyzing and visualizing the expression of the prognostic model FRGs between the tissue of normal and IPF by using the “limma” and “beeswarm” package of R. The value of *p* < 0.05 was considered statistically significant.

### Statistical Analysis

The Perl, R (4.0.1), and SPSS 18.0 were used for data processing and statistical analysis. The Wilcoxon and univariate Cox regression tests were utilized to evaluate the expression levels of FRGs in BALF between IPF and healthy controls and identified the genes associated with prognosis, respectively. A prognostic model was generated by the LASSO-penalized Cox regression analysis and further evaluated the predictive capacity of which by K–M survival and ROC curve analysis. The univariate and multivariate Cox regression analysis were utilized to identify independent predictors of OS. The probability *p* < 0.05 was considered significant.

## Results

### Identification of FRGs in IPF

**Figure 1:**
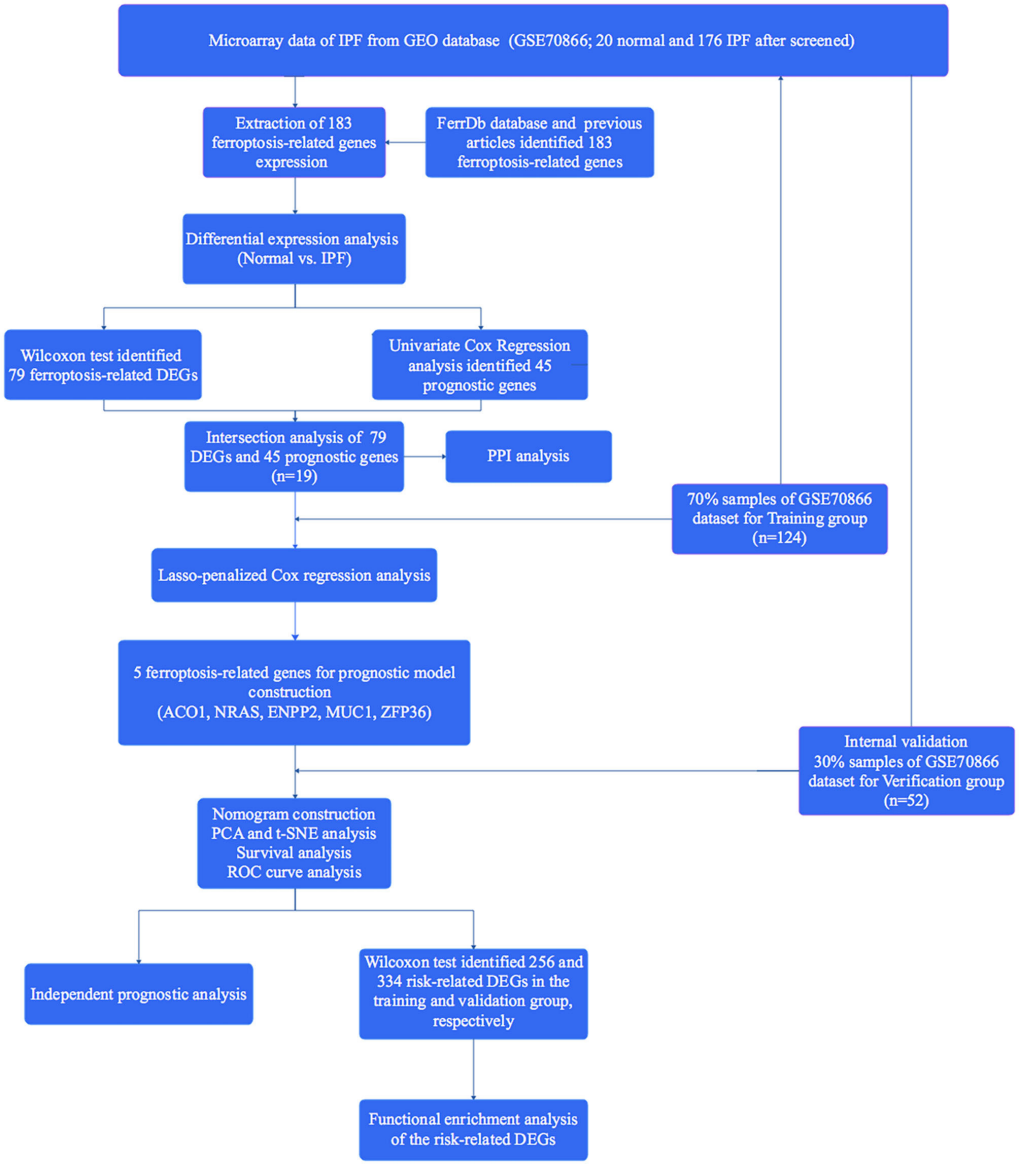
Flow diagram of the present study.

**Figure 2:**
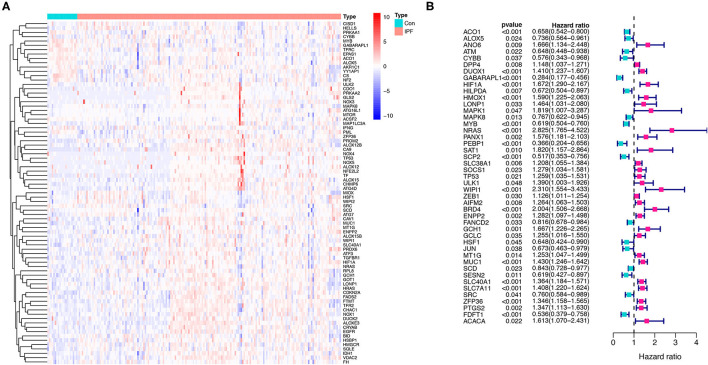
Identification of the FRGs in the patients with IPF from the Gene Expression Omnibus (GEO) database. **(A)** The expression of the 79 ferroptosis-related DEGs in IPF. **(B)** The 45 ferroptosis-related prognostic genes in IPF. *p* < 0.05 was considered significant. FRGs, ferroptosis-related genes; IPF, idiopathic pulmonary fibrosis; and DEGs, differentially expressed genes.

**Figure 3:**
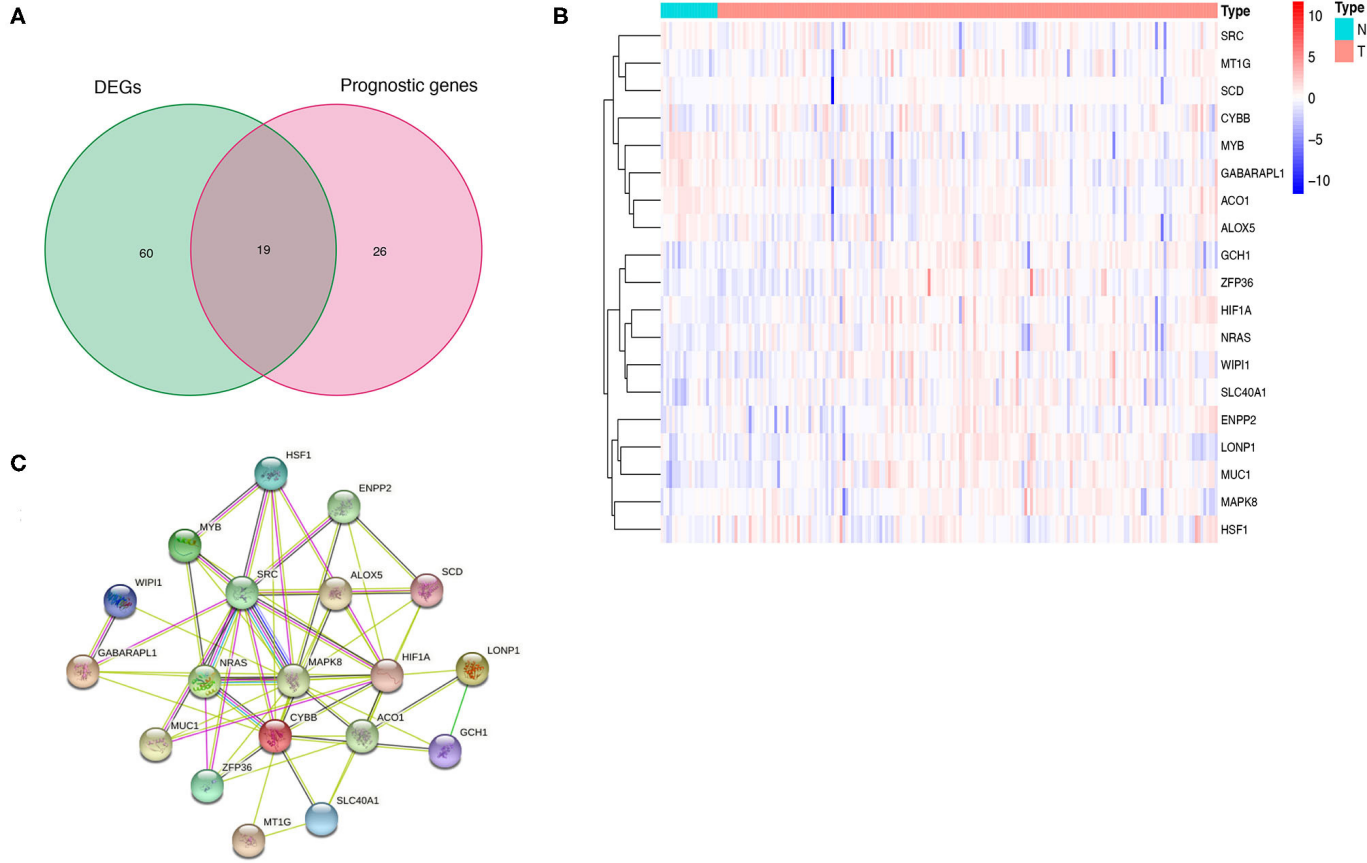
Identification and expression of the overlapping genes between DEGs and prognostic genes in IPF. **(A)** Venn diagram of 19 overlapping genes. **(B)** Expression of the 19 overlapping genes in IPF. **(C)** The correlation network of the overlapping genes. IPF, idiopathic pulmonary fibrosis; DEGs, differentially expressed genes; PPI, protein–protein interaction.

A total of 176 patients with IPF and 20 healthy controls from the cohort of GSE70866 dataset were enrolled in this study, and 183 FRGs were explored from the FerrDb database and the literature report (Supplementary Table 1). The detailed information of the candidate patients is presented in Supplementary Table 2. Figure 1 illustrates the flow of our research. In the present study, we found that 79 FRGs (43.17%) were significantly differentially expressed in BALF between IPF and normal population (Figure 2A), and 45 (24.59%) FRGs were associated with OS of IPF (Figure 2B). In addition, 19 ferroptosis-related prognostic DEGs were identified using the Venn diagram (all *P* < 0.05, Figures 3A,B).

### PPI Network Construction Based on Prognostic FRGs

The PPI network based on the 19 prognostic ferroptosis-related DEGs was generated by the STRING online platform (Figure 3C), which demonstrated that there is a significant correlation between these FRGs in BALF of IPF.

### Construction of a Prognostic Model in the Training Group

**Figure 4:**
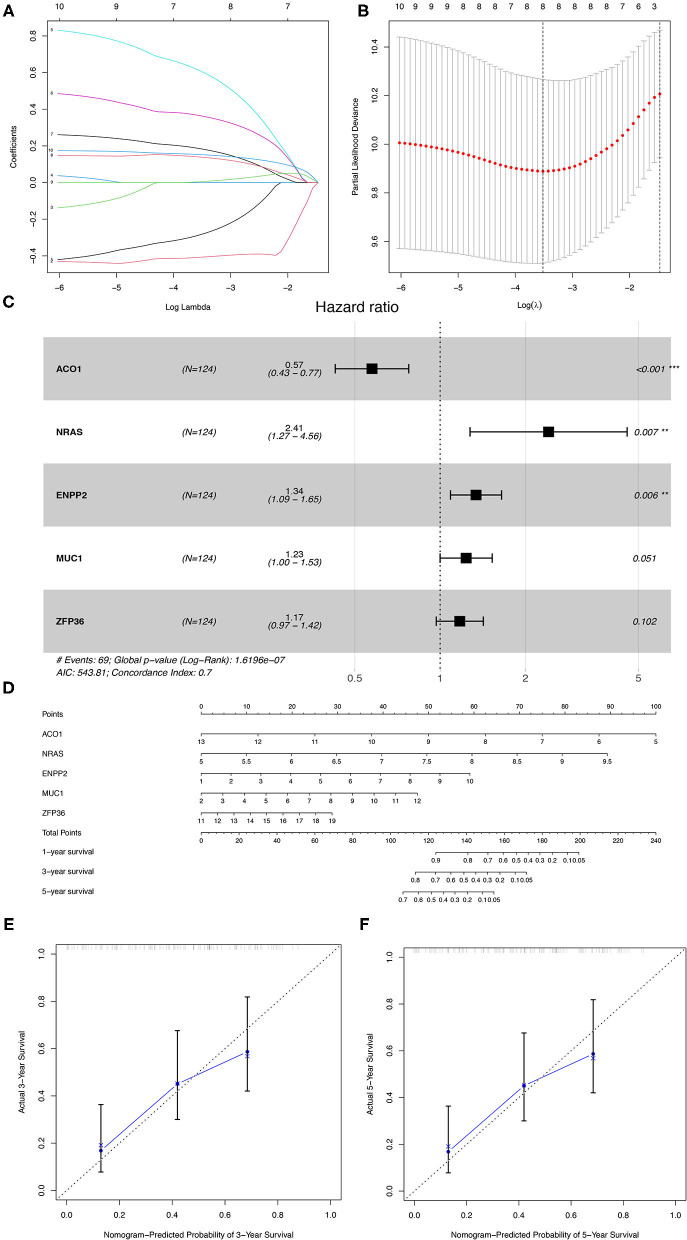
The construction of the prognostic model of IPF in the training group. **(A)** LASSO coefficients profiles of the 19 overlapping genes. **(B)** LASSO regression with 10-fold cross-validation obtained eight prognostic FRGs. **(C)** The multivariate Cox regression analysis identified five prognostic FRGs for prognostic model construction. **(D)** The nomogram of the prognostic model based on the five FRGs. The calibration curves of the nomogram for predicting 3- **(E)** and 5-years survival **(F)** of the patients with IPF. IPF, idiopathic pulmonary fibrosis; FRGs, ferroptosis-related genes. *P < 0.05, **P < 0.01, ***P < 0.001.

The 176 patients with IPF in the GSE70866 dataset were randomly categorized into training and testing cohorts at a ratio of 7:3, and the LASSO-penalized Cox regression analysis was used to set up a prognostic signature in the training group. The result showed that only five genes (aconitase 1 [ACO1], neuroblastoma RAS viral (v-ras) oncogene homolog [NRAS], ectonucleotide pyrophosphatase/phosphodiesterase 2 [ENPP2], mucin 1 [MUC1], and ZFP36 ring finger protein [ZFP36]) remained in LASSO regression from the 19 prognostic FRGs (Figures 4A–C), among which, NRAS, ENPP2, MUC1, and ZFP36 are the risk factors, and ACO1 is a protective factor for IPF prognosis (Figure 4C, Table 1). Then, the nomogram of five FRGs for predicting 1-, 3-, and 5-year OS of IPF was visualized in Figure 4D. At the same time, the calibration curves showed satisfactory agreement between the predicted and observed values at the probabilities of 3- and 5-year survival (Figures 4E,F). These results revealed a good accuracy of the nomogram in predicting the 3- or 5-year survival in patients with IPF.

**Table 1:** Multivariate Cox regression analysis of the five FRGs.

**Gene**	**Description**	**coef**	**HR(95%CI)**	***P*-value**
ACO1	Aconitase 1, soluble	−0.553	0.575 (0.427,0.775)	<0.001
NRAS	Neuroblastoma RAS viral (v-ras) oncogene homolog	0.880	2.412(1.275,4.564)	0.007
ENPP2	Ectonucleotide pyrophosphatase/phosphodiesterase 2	0.291	1.338(1.087,1.646)	0.006
MUC1	Mucin 1, cell surface associated	0.211	1.235(0.999,1.526)	0.051
ZFP36	ZFP36 ring finger protein	0.159	1.173 (0.969,1.420)	0.102

*FRGs, ferroptosis-related genes; coef, regression coefficients; HR, hazard ratio; 95% CI, 95% confidence interval*.

**Figure 5:**
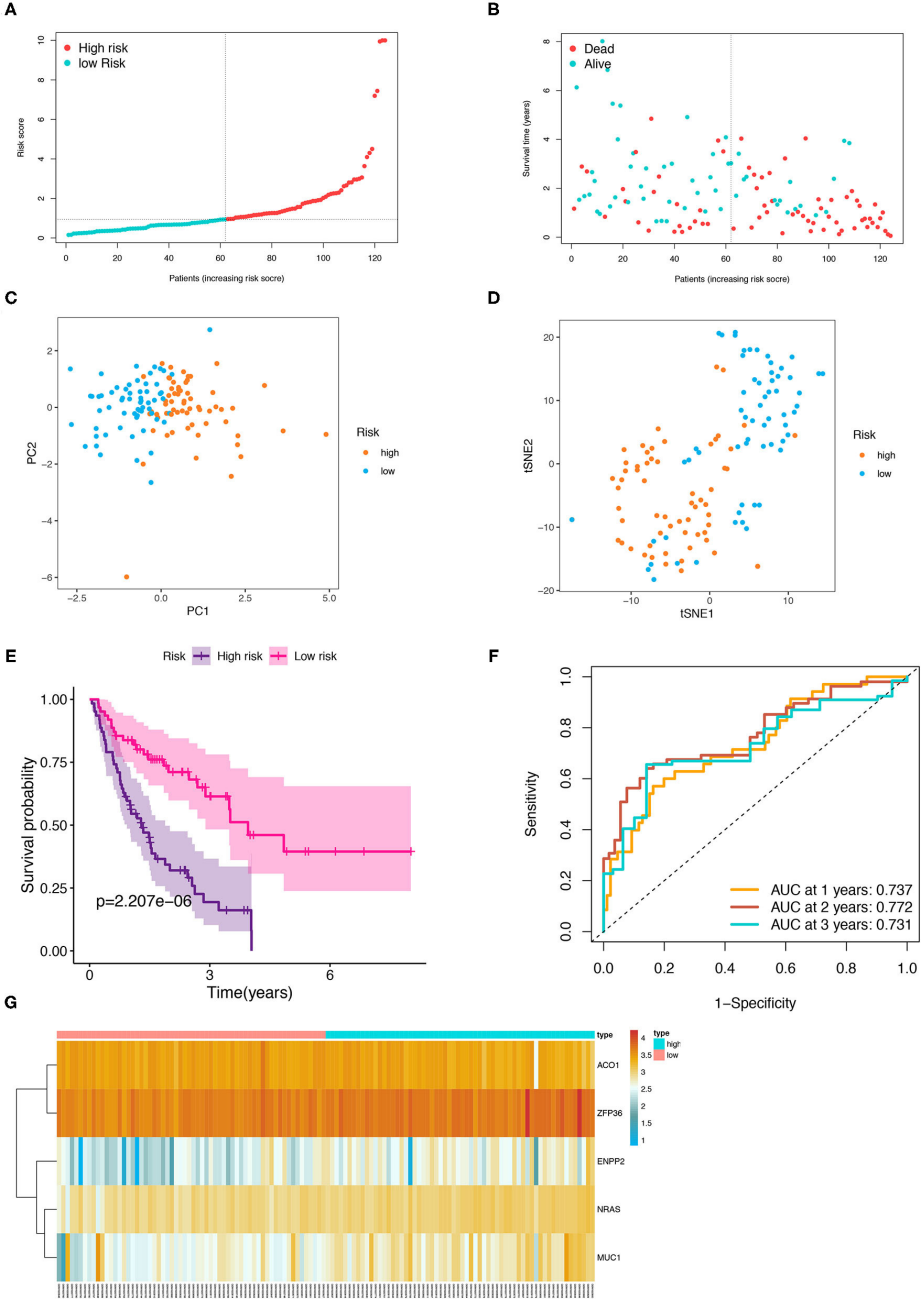
Prognostic analysis of the five-FRGs signature in the training group. Distribution of IPF samples **(A)** and OS status **(B)** based on the median risk score. PCA **(C)** and t-SNE **(D)** analysis of the training group. Kaplan–Meier survival **(E)** and time-dependent ROC curve **(F)** analysis of the five-FRGs signature in training group. **(G)** Heatmap of the expression profiles of the five-FRGs in low- and high-risk groups. FRGs, ferroptosis-related genes; IPF, idiopathic pulmonary fibrosis; OS, overall survival; PCA, principal component analysis; t-SNE, t-distributed stochastic neighbor embedding; ROC, receiver operating characteristic.

Then, the patients with IPF in the training cohort were categorized into a high- and low-risk group according to the median risk score (Figure 5A), and the high-risk group was demonstrated to be more likely to encounter death earlier (Figure 5B). At the same time, the patients in both subgroups were demonstrated to be distributed in a discrete direction by PCA and t-SNE analysis (Figures 5C,D). Subsequently, the predictive power of the prognostic model was further evaluated by the survival and a ROC curve analysis. The K–M plot revealed that the patients with IPF in the high-risk group had a worse prognosis than those in the low-risk group (*p* < 0.001, Figure 5E). In addition, the time-dependent ROC curve analysis elucidated that the 1-, 2-, and 3-year area under the curve (AUC) were 0.737, 0.772, and 0.731, respectively (Figure 5F). The expression levels of the five FRGs in each sample between the two risk groups are presented in Figure 5G. In addition, three of the five FRGs (NRAS, MUC1, and ZFP36) were also found differentially expressed between the normal and IPF tissue in the combination of the four GEO datasets (Supplementary Figure 1, *p* < 0.05).

### Validation of the Prognostic Signature in the Testing Group

**Figure 6:**
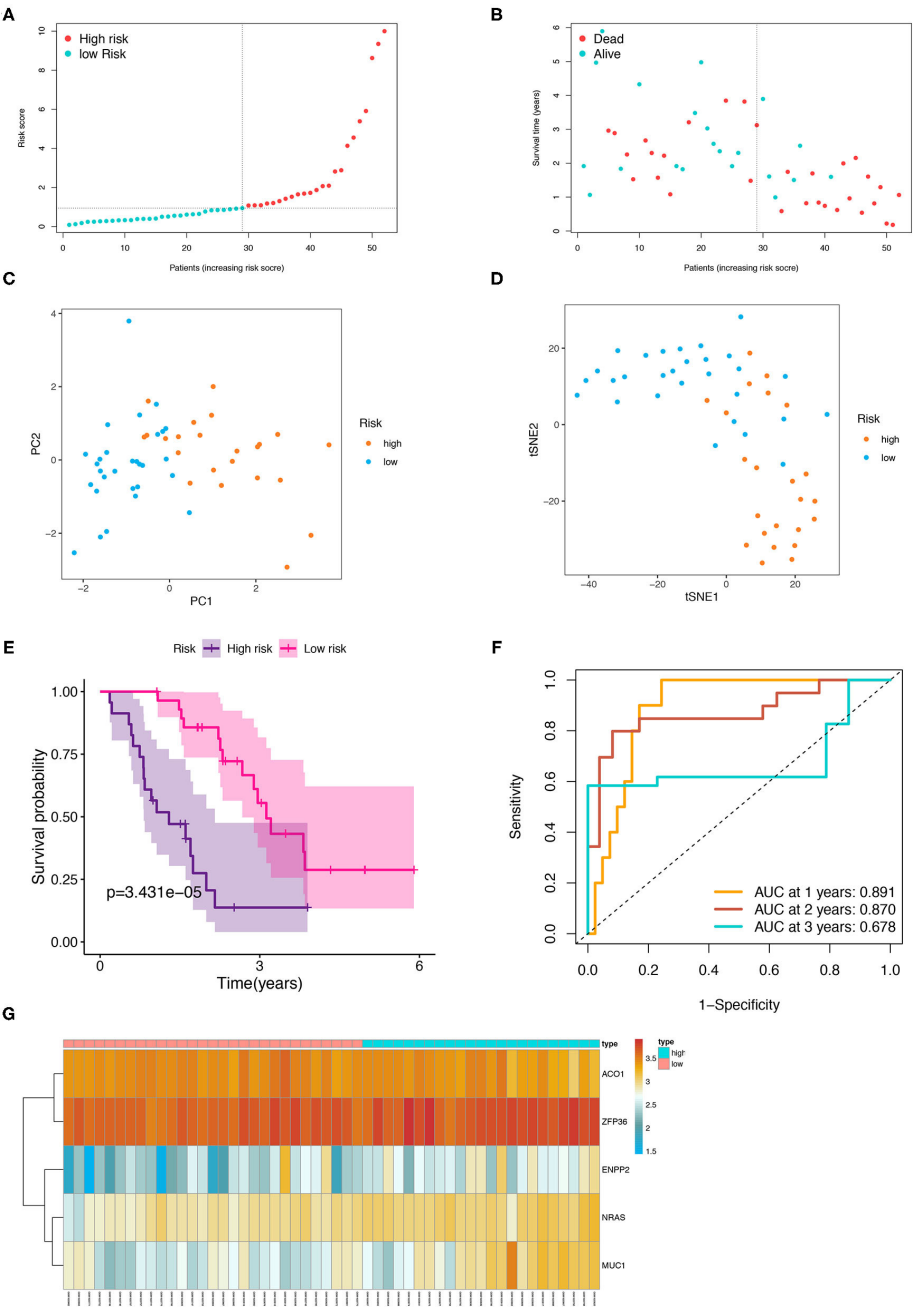
Internal validation of the five-FRGs signature. Distribution of IPF samples **(A)** and OS status **(B)** based on the median value of the risk scores. PCA **(C)** and t-SNE **(D)** analysis of the testing group. Kaplan–Meier survival **(E)** and time-dependent ROC curve **(F)** analysis of the five-FRGs signature in the testing group. **(G)** Heatmap of the expression profiles of the five-FRGs in low- and high-risk groups of the testing cohort. FRGs, ferroptosis-related genes; IPF, idiopathic pulmonary fibrosis; OS, overall survival; PCA, principal component analysis; t-SNE, t-distributed stochastic neighbor embedding; ROC, receiver operating characteristic.

The patients from the testing cohort were also separated in the two risk groups based on the median risk score to evaluate the robustness of the prognostic model constructed in the training cohort (Figure 6A). Similar to the results of the training cohort, the patients with the high-risk tended to reach the end of life earlier in the validation group (Figure 6B). The PCA and t-SNE analysis showed that the distribution in the discrete direction of patients in the two subgroups was similar to that in the training group (Figures 6C,D). Meanwhile, a worse prognosis was also found in the patients with IPF with high-risk in the testing group (*p* < 0.001, Figure 6E), and an ROC curve also elucidated a satisfactory accuracy of the prognostic model to identify the 1-, 2-, and 3-year OS of IPF in the testing group (Figure 6F, AUC at 1 year: 0.891; 2 years: 0.870; and 3 years: 0.678). The heatmap showed that the expression trend of the five FRGs between the high- and low-risk samples of the testing group was similar to that of the training group (Figure 6G).

### Independent Prognostic Analysis

**Figure 7:**
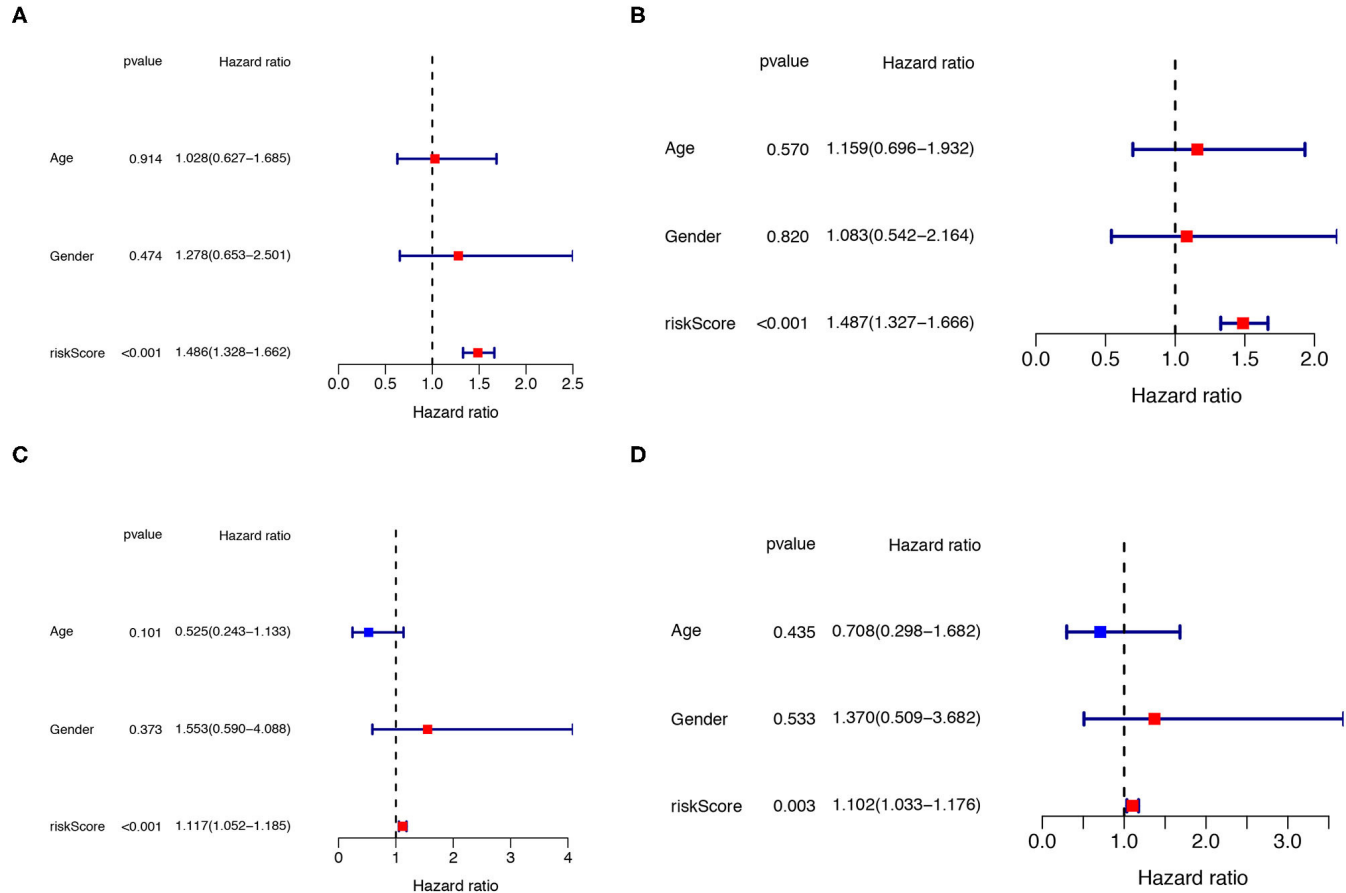
Independent prognostic analysis of clinical parameters and risk score. The univariate **(A)** and multivariate **(B)** Cox regression analysis of the associations between the risk score, the clinical parameters and OS of IPF patients in the training cohort. The univariate **(C)** and multivariate **(D)** Cox regression analysis of the associations between the risk score, clinical parameters and OS of IPF patients in the testing cohort. IPF, idiopathic pulmonary fibrosis; OS, overall survival.

The univariate and multivariate Cox regression analyses were performed to determine whether the clinical parameters (such as, gender and age; the cut-off value of age is 65 years) and risk score are independent prognostic factors for OS in IPF. The results demonstrated that the risk score remained as an independent risk factor for the outcome of IPF in both the training (Figures 7A,B) and testing groups (Figures 7C,D) after controlling for gender and age.

### Functional Enrichment Analysis

**Figure 8:**
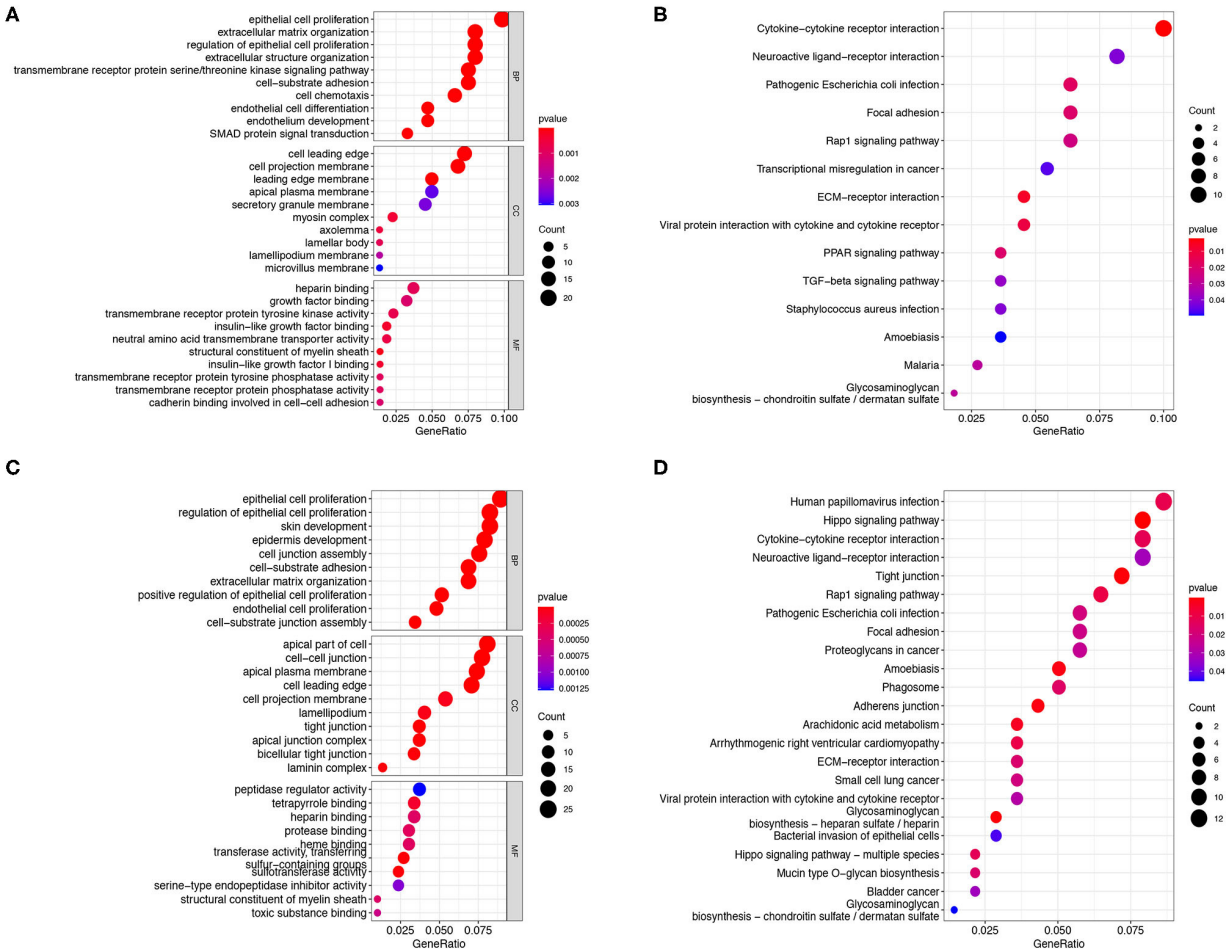
GO and KEGG analysis. The GO enrichment and KEGG pathways of the risk score-related DEGs in the training cohort **(A,B)** and testing cohort **(C,D)**. GO, Gene Ontology; KEGG, Kyoto Encyclopedia of Genes and Genome; DEGs, differentially expressed genes.

There were 256 and 334 risk-related DEGs identified in the training and validation group by using Wilcoxon's test, respectively (Supplementary Table 3). The GO and KEGG analysis on the risk-related DEGs were performed in the present study to further investigate the potential functions and pathways that are associated with the risk score. The results indicated that the risk-related DEGs mainly focused on epithelial cell proliferation, SMAD protein signal transduction, extracellular matrix organization, cell projection membrane, growth factor binding, cadherin binding involved in cell–cell adhesion, etc., in the training group (Figure 8A). In addition, except for some functions mentioned above, the risk-related DEGs were also found to be enriched in the regulation of cell-junction, tight junction, and protease binding in the validation group (Figure 8C). KEGG pathway analysis also indicated that the ECM-receptor interaction, Rap1 signaling pathway, focal adhesion, and cytokine-cytokine receptor interaction pathway were enriched in both the cohorts (Figures 8B,D). In addition, the transforming growth factor-beta (TGF-β) signaling pathway was also identified enriched in the training cohorts (Figure 8B).

## Discussion

Idiopathic pulmonary fibrosis is a progressive and devastating disease with limited treatment options, unsatisfactory therapeutic effects, and poor prognosis (1, 2). Despite significant innovations in the treatment and diagnosis of IPF compared with the past, medication has not enhanced the survival rate. Early assessment of disease progression and prognosis, and timely appropriate treatment have important clinical significance due to the variable and unpredictable of the clinical course of IPF (36). Hence, there is a current and urgent need for reliable, safe, and feasible markers that can accurately predict prognosis, to make the management of IPF patients more accurate, personalized, and timely.

Ferroptosis is considered as an effective cancer treatment because it overcomes the resistance of malignant cells to chemotherapy and promotes the clearance of defective cells. Recently, various studies have illustrated that ferroptosis regulate the development of pulmonary fibrosis to some extent (24–27, 37), however, as the most common pathological type of pulmonary fibrosis, the correlation between ferroptosis and the OS of patients with IPF has not been investigated. In this study, we found that approximately half of FRGs (79/183, 43.17%) in BALF were differentially expressed between IPF and healthy controls, and 19 of them were correlated with the OS of the IPF patients. In addition, a novel prognostic model integrating the five FRGs was first established and verified in an internal cohort. Then, we further verified that the risk score is an independent prognostic factor for OS of IPF and revealed the potential function and pathways that are associated with the risk score-related DEGs by the functional enrichment analysis. The findings showed above strongly indicate a potentially important role of ferroptosis in IPF.

In our study, the predictive model was established based on the five FRGs (ACO1, NRAS, ENPP2, MUC1, and ZFP36). Cytosolic aconitase 1/iron regulatory protein 1 (ACO1/IRP1) is a bifunctional protein expressed in cytoplasm that performs its function as aconitase or regulating intracellular iron homeostasis according to iron availability (38, 39). Wang J et al. found that IRP1/ACO1 activated by nitric oxide enables impair cellular iron homeostasis and triggers iron accumulation during neuroinflammation, leading to neuronal death (40). In the present study, we elucidated that ACO1 is downregulated in patients with IPF and as a protective factor. NRAS (N-ras), a member of the RAS family, and commonly mutated in almost all human cancers (41). NRAS has been verified associated with the erastin resistance in hepatocellular carcinoma (42), however, whether ferroptosis pathway is involved in this effect needs to be further clarified. N-Ras was also found to promote renal fibrosis by participating in TGF-β1 induced proliferation and the synthesis of collagen and fibronectin (43), whereas the function of NRAS in IPF has not been studied. We found that the overexpressed NRAS is associated with the poor prognosis in IPF. ENPP2 encodes the protein autotaxin (ATX), an enzyme primarily responsible for the production of extracellular lysophosphatidic acid, has been illustrated to protect cardiomyocytes from ferroptosis induced by erastin (44). In contrast, ENPP2/ATX was found to be upregulated and promote the development of fibrosis in the BALF of pulmonary fibrosis patients (45). Similar to the results of the previous study (45), we found that ENPP2 is upregulated in the BALF of IPF and associated with poor prognosis. Hence, it may be doubtful whether ENPP2 is involved in the pathogenesis of IPF through regulating ferroptosis. Mucin 1 (MUC1) is a glycoprotein and abnormally overexpressed in most respiratory diseases, such as IPF (46), which was also found involved in the stabilization of the system X${}_{\text{C}}^{-}$ cystine/glutamate antiporter (xCT) complex, an important intracellular antioxidant element involved in the ferroptosis (47, 48) and in breast cancer cells (49). Herein we elucidated the potential risk role of FRGs MUC1 in the IPF prognosis. ZFP36 ring finger protein has two tandem repeat CCCH zinc fingers domains, which mainly regulate the fate of cytoplasmic mRNA and modulate the response of the cell to oxidative stress, apoptosis, and lipid peroxidation by affecting the post-transcriptional effects on specific target mRNAs (50, 51). Overexpression of ZFP36 was found to result in resistance to ferroptosis by triggering autophagy inactivation and blocking autophagy ferritin degradation (52). Interestingly, ZFP36 was identified as a specific and shared biological biomarker in COPD, asthma, and IPF (53), and was verified as a risk-predicting factor in our study. In summary, all the five FRGs were found to be upregulated in BALF of IPF and were correlated with unfavorable prognosis in our study except for ACO1. However, whether these genes play a role in the prognosis of patients with IPF by affecting the ferroptosis process need to be further clarified.

It is worth mentioning that we further evaluated the expression of the five FRGs in the tissue of IPF. The results demonstrated that three of the five FRGs (NRAS, MUC1, and ZFP36) are dysregulated in the lung of IPF patients. Contrary to our findings, MUC1 and ZFP36 were found to be downregulated in the lung tissues of patients with IPF. The possible reasons are as follows: First, the authors of the IPF-BALF dataset (GSE70866) pointed that none of the patients received pirfenidone or nintedanib before BAL examination in their study (54). However, all the lung tissue of the GSE110147, GSE72073, GSE53845, and GSE24206 datasets were obtained from the patients with IPF at the time of biopsy or lung transplantation, and whether patients received treatment were not mentioned in the literature (55–58). It could be that the drug treatment changes the expression of these genes. The anti-fibrotic effects of pirfenidone are proved that partially mediated by the inhibition of MUC1 bioactivation (59). Second, the sample size of the tissue datasets (28 normal and 84 IPF) is less than the BALF dataset (28 normal and 176 IPF), which could also contribute to the difference in the results. Third, the gene expression levels were also related to race, tissue sampling site, disease severity, and complications. Therefore, more rigorous studies need to be designed to determine the expression of these genes in lung tissue and BALF, as well as the mechanism of their involvement in the occurrence and development of pulmonary fibrosis.

Function analysis found that the risk score-related DEGs are mainly enriched in epithelial cell proliferation and extracellular matrix organization, etc. In addition, these DEGs were identified enriched in the cytokine–cytokine receptor interaction, TGF-β signaling pathways, focal adhesion, and ECM–receptor interaction signaling pathways, all of them are the most crucial pathways involved in the development of IPF (60–62). These results indicated that the FRGs may regulate the development and progression of IPF by modulating these major pathways.

The advantage of this study is that the FRGs were explored more comprehensively by using the FerrDb database and the previous studies. In addition, the data of IPF from an online open database were systematically analyzed and the predicting value about the FRGs in IPF prognosis was evaluated and summarized for the first time. Meanwhile, there are some limitations present in this study. First, the sample size of normal control from the GEO database was not large enough. Second, only internal validation of the model was performed in the study due to the lack of similar datasets available for external validation. Third, molecular biology experiments were not carried out to further investigate the above results. Therefore, the prospective cohort study and molecular biology experiments need to be designed and performed to further verify the accuracy of the prognostic model and the mechanism of the five FRGs in the pathogenesis of IPF.

## Conclusion

To sum up, 19 FRGs associated with the OS were identified in BALF of IPF and a novel prognostic model was constructed based on the five FRGs in the present study. Then, the model was verified as an independent risk factor of OS of IPF patients in both the derivation and validation cohorts. These findings have potential reference value for guiding the treatment and prognosis evaluation of the patients with IPF.

## Data Availability Statement

Publicly available datasets were analyzed in this study. This data can be found here: https://www.ncbi.nlm.nih.gov/geo/.

## Author Contributions

HR, MC, and ML: conception and design. HR and MC: administrative support. ML, KW, YZ, MF, AL, JZ, TY, PS, DL, and GZ: provision of study materials or patients. ML, KW, YZ, MF, AL, JZ, and DL: experiments operations, collection, and assembly of data. ML, KW, YZ, AL, JZ, TY, PS, DL, GZ, MC, and HR: data analysis and interpretation. All authors: manuscript writing. All authors contributed to the article and approved the submitted version.

## Funding

This study was funded by the Key Research and Development Projects of Shaanxi Province (Nos. 2018SF-071, 2019KW-034, 2018KW-039, and 2021SF-041), Fundamental Research Funds for the Central Universities in Xi'an Jiaotong University (Nos. xzy012021064 and 1191320149); the National Natural Science Foundation of China (No. 81802291), China Postdoctoral Science Foundation Grant (No. 2019M653666).

## Conflict of Interest

The authors declare that the research was conducted in the absence of any commercial or financial relationships that could be construed as a potential conflict of interest.

## Publisher's Note

All claims expressed in this article are solely those of the authors and do not necessarily represent those of their affiliated organizations, or those of the publisher, the editors and the reviewers. Any product that may be evaluated in this article, or claim that may be made by its manufacturer, is not guaranteed or endorsed by the publisher.

## Abbreviations

IPF: Idiopathic pulmonary fibrosis
ILD: Interstitial pulmonary disease
COPD: Chronic obstructive pulmonary disease
BALF: Bronchoalveolar lavage fluid
FRGs: Ferroptosis-related genes
GEO: Gene Expression Omnibus
DEGs: Differentially expressed gene
LASSO: Least absolute shrinkage and selection operator
PCA: Principal component analysis
t-SNE: t-distributed stochastic neighbor embedding
K-M: Kaplan–Meier
ROC: receiver operational characteristic
AUC: Area under curve
OS: Overall survival
GO: Gene Ontology
KEGG: Kyoto Encyclopedia of Genes and Genomes
PPI: Protein-protein interactions
ACO1, Aconitase 1: soluble
NRAS: Neuroblastoma RAS viral (v-ras) oncogene homolog
ENPP2: Ectonucleotide pyrophosphatase/phosphodiesterase 2
MUC1, Mucin 1: cell surface associated
ZFP36: ZFP36 ring finger protein
ATX: autotaxin.
