# Supplementary material for: Ferroptosis-Related Genes in Bronchoalveolar Lavage Fluid Serves as Prognostic Biomarkers for Idiopathic Pulmonary Fibrosis
Source: Front Med (Lausanne). 2021 Oct 4;8:693959. doi: 10.3389/fmed.2021.693959 (PMC8520927; doi:10.3389/fmed.2021.693959)
Supplement: Supplementary file 1 [file Data_Sheet_1.docx]

Supplementary Table 1 183 FRGs were identified by FerrDb database and previous studies.

| **ABCC1** | **ACO1** | **ACSF2** | **ACSL4** | **ACVR1B** | **ALOX12B** | **ALOX12** | **ALOX15B** | **ALOX15** | **ALOX5** |
| --- | --- | --- | --- | --- | --- | --- | --- | --- | --- |
| ALOXE3 | ANO6 | ATF3 | ATG13 | ATG16L1 | ATG3 | ATG4D | ATG5 | ATG7 | ATM |
| ATP5MC3 | BACH1 | BAP1 | BECN1 | BID | CDKN2A | CARS1 | CDO1 | CHAC1 | CS |
| CYBB | DNAJB6 | DPP4 | DUOX1 | DUOX2 | EGFR | EGLN2 | ELAVL1 | EMC2 | EPAS1 |
| FBXW7 | FLT3 | G6PD | G6PDX | GABARAPL1 | GABARAPL2 | GLS2 | GOT1 | HIF1A | HILPDA |
| HMGB1 | HMOX1 | HRAS | IDH1 | IFNG | LINC00472 | KEAP1 | KRAS | IREB2 | LONP1 |
| LPCAT3 | LPIN1 | MAPK14 | MAPK1 | MAP1LC3A | MAPK3 | MAPK8 | MAPK9 | MIOX | MIR6852 |
| MTDH | MYB | NCOA4 | NOX1 | NOX3 | NOX4 | NOX5 | NRAS | PANX1 | PEBP1 |
| PGD | PHKG2 | PIK3CA | PRKAA1 | PRKAA2 | RPL8 | SAT1 | SCP2 | SIRT1 | SLC1A5 |
| SLC38A1 | SNX4 | SOCS1 | TAZ | TF | TFR2 | TFRC | TGFBR1 | TLR4 | TNFAIP3 |
| TP53 | ULK1 | ULK2 | VDAC2 | WIPI1 | WIPI2 | YY1AP1 | ZEB1 | ACSL3 | AIFM2 |
| AKR1C1 | AKR1C2 | AKR1C3 | ARNTL | ATF4 | BRD4 | CA9 | CAV1 | CBS | CD44 |
| CDKN1A | CHMP5 | CHMP6 | CISD1 | CISD2 | ENPP2 | FADS2 | FANCD2 | Fer1HCH | FH |
| FTH1 | FTMT | GCH1 | GCLC | GPX4 | HELLS | HSF1 | HSPA5 | HSPB1 | ISCU |
| JUN | LAMP2 | MIR17 | MIR137 | LINC00336 | MIR212 | MIR9-1 | MIR9-2 | MIR9-3 | MT1G |
| MTOR | MUC1 | NF2 | NFE2L2 | NFS1 | NQO1 | OTUB1 | PLIN2 | PML | PRDX6 |
| PROM2 | RB1 | SCD | SESN2 | SLC3A2 | SLC40A1 | SLC7A11 | SQSTM1 | SRC | STAT3 |
| TMBIM4 | TP63 | ZFP36 | GCLM | GSS | HMGCR | CRYAB | PTGS2 | FDFT1 | HSBP1 |
| STEAP3 | STEAP3 | STEAP3 |  |  |  |  |  |  |  |

Supplementary Table 2 The clinical information of the GSE 70866 cohort

| Clinical features | Control (n=20) | IPF(n=176) |
| --- | --- | --- |
| Age | 61.25 ± 8.32 | 68.07 ± 9.52 |
| <65 | 12 (60.0%) | 59 (33.5%) |
| ≥65 | 8 (40.0%) | 117(66.5%) |
| Gender |  |  |
| Female | 4( 20.0%) | 32 (18.2%) |
| Male | 16 (80.0%) | 144 (81.8%) |
| Survival status |  |  |
| Alive | 20 (100.0%) | 76 (43.2%) |
| Dead | 0 (0.0%) | 100 (56.8%) |

IPF, idiopathic pulmonary fibrosis.
